# Impact of the COVID-19 Pandemic on the Dental Preferences of Patients in the Private Sector

**DOI:** 10.3390/ijerph19042183

**Published:** 2022-02-15

**Authors:** Klaudia Migas, Michał Marczak, Remigiusz Kozłowski, Andrzej Kot, Anna Wysocka, Aleksandra Sierocka

**Affiliations:** 1 Department of Management and Logistics in Healthcare, Medical University of Lodz, 90-419 Lodz, Poland; klaudia.migas@stud.umed.lodz.pl (K.M.); michal.j.marczak@gmail.com (M.M.); kott22@gmail.com (A.K.); 2 Center of Security Technologies in Logistics, Faculty of Management, University of Lodz, 90-237 Lodz, Poland; remigiusz.kozlowski@umed.lodz.pl; 3 Department of Internal Medicine in Nursing, Faculty of Health Sciences, Medical University of Lublin, 20-093 Lublin, Poland; annawysocka@umlub.pl

**Keywords:** COVID-19, COVID-19 dentistry, COVID-19 pandemic

## Abstract

Proper oral health care has an impact on the health of the entire body. The COVID-19 pandemic has affected the functioning of the healthcare sector, including dental services. The aim of this study was to analyse the behaviour of patients with regard to their use of dental services during the pandemic. The data were obtained from paper registration forms taken from five dental offices in the city of Cracow between March 2019 and February 2021. During the analysed periods, interest among first-time patients in dental services decreased to 37% (during the month when interest in dental services was at its lowest) compared to the year preceding the COVID-19 pandemic. The number of cancelled visits increased by between 15% and 50% compared to the pre-pandemic period. During the pandemic, appointments made by existing patients increased by up to 84% compared to 2019. The decision by patients to postpone dental treatment not only has adverse effects on their oral and body health, but in turn results in higher health care costs. Given the potential for another pandemic, further long-term research is required to develop and implement special protocols to make the public aware of the safety of health care.

## 1. Introduction

The COVID-19 pandemic has exerted an impact on healthcare worldwide. In response to the health emergency governments and the WHO (World Health Organization) have issued recommendations and guidelines regarding the functioning of health care, including in the private sector and dental care [1,2,3,4].

The high risk of infection for both dental office staff and patients led to the implementation of a number of procedures and practices aimed at minimizing this risk, including the use of personal protective equipment, measuring body temperature, conducting epidemiological interviews, air disinfection, extending the time interval between patients and rotating the work of dental offices [2,3,4,5,6].

At the same time, the incomplete knowledge of the nature of COVID-19, the specificity of the SARS-CoV-2 virus and infections, as well as the various restrictions introduced by governments, have given rise to patient uncertainty a shift to decreased interest in specific dental services [3].

The aim of this study was to assess the degree of patients’ interest in dental services during the COVID-19 pandemic, in particular in the period from March 2020 to February 2021, and to compare this interest with patients’ interest in dental services in the preceding year, i.e., from March 2019 to February 2020, focusing on five dental offices in the Polish city of Cracow.

According to the data of the Central Statistical Office, 1584 dentists work in Cracow, which is 2 dentists per 1000 inhabitants of Cracow. In Cracow, out of 1584 dentists, 1318 (83.2%) are general dental practitioners (GDP) and 266 (16.8%) are dental specialists. A total of 42,425 dentists work in Poland, which is 1 dentist per 1000 inhabitants of Poland. General dental practitioners (GDP) account for 85.6% (36,293) of all dentists in Poland and dental specialists account for 14.4% (61,320) of all dentists in Poland.

According to Eurostat data, in European countries such as Germany and Norway the number of dentists is 8 per 1000 inhabitants.

According to the data of the Central Statistical Office, there are 4326 dental offices in Poland, and 224 dental offices in Cracow, which accounts for 5.2% of the Polish dental market.

In five dental offices qualified for the study, a full range of dental services is provided, and for every dental specialization, these offices have their own prosthetic laboratory, X-ray and computed tomography diagnostics, and 386 dentists work there (316 general dental practitioners and 70 dental specialists), which constitutes 24% of working dentists in Cracow. 

According to data from the Statistical Office in Cracow, in 2019 Cracow had a total population of 779,115 inhabitants. According to the data of the Central Statistical Office, in 2020 the population of the city of Cracow was 779,966 people, including 416,195 women. The unemployment rate was 3.1%. In 2019, the population was 779,115 people, including 415,628 women, and the unemployment rate was 2.0%. The unemployment rate in Poland in 2019 was 5.2%, and in 2020 it was 6.2%. In 2019, the population of Poland was 38,383,000, including 19,800,000 women, and in 2020 the population of Poland was 38,265,000, including 19,762,772 women.

In the future, as a result of migration, the population is expected to increase both in the city itself and in the surrounding municipalities. Demographic trends indicate a shift towards a higher share of females in the total population together with a high share of post-working age residents. Cracow has an aging population and a declining birth rate. The total number of females registered as permanent residents in Cracow will decline at a faster rate (23,500 people) than males registered as permanent residents (17,800 people). The reason for this discrepancy will be a more rapid increase in average life expectancy in men than in women. However, significant differences in terms of average life expectancy will continue to exist between women and men in Cracow (74 years for men and 81 years for women in 2023). Expected demographic trends in Cracow include a decline in the population as a whole and changes in the age structure.

The most important negative trend will be a significant acceleration in the aging of the population during the forecast period. The post-working age population (i.e., 60 and above for women and 65 and above for men) will be replenished with members of the post-war baby boomer generation. According to forecasts, the share of elderly permanent residents in the city will increase to 28.4% of the total population in 2023. The total number of permanent residents is expected to increase by up to 52,500. More women will move to the city as a consequence of the development of the city’s service sector. The dominant group of migrants will be people aged 20–35, while in other age groups an outflow of the population to suburban areas is forecast. The population of 40–59-year-olds is expected to stabilize. 

According to the demographic forecasts of the Statistical Office, by 2035 Cracow’s population will increase to 800,000, making it one of the few cities in Poland with a positive growth trend—the forecast for other voivodship capitals is more negative.

## 2. Materials and Methods

1. The research focused on 5 dental offices in the Polish city of Cracow and was based on paper questionnaires prepared specifically for the study. The data recorded by dental offices were in accord with the medical services they provide and regulated by the relevant legislation. The questionnaire was tested before the start of the study on a population of 311 people. A satisfactory result was the intra-class correlation coefficient (ICC) > 0.80, when the obtained result was lower than satisfactory, the given item was edited.

Five dental offices were qualified for the study, which provided a full range of dental services, all specializations, had their own prosthetic laboratory and their own X-ray diagnostics and 3D computed tomography laboratory, and provided dental services continuously throughout the COVID-19 pandemic. Dental offices that did not provide services in all dental specialties and did not have their own prosthetic laboratories and X-ray diagnostics were excluded from the study. The exclusion of such units from the study allowed for the exclusion of factors influencing patients’ treatment decisions, which could be influenced by limitations in accepting orders by prosthetic laboratories and delays in prosthetic laboratories, as well as limitations in admitting patients to X-ray diagnostic tests. As a result, the factor constituting a given dental facility in relation to the patient’s preferences was excluded.

Qualified units accounted for 25% of Cracow dental clinics, providing dental services of all specialties, having their own 2D and 3D X-ray diagnostics laboratory and a prosthetic laboratory. Selected dental offices can be considered representative of the city of Cracow.

The data covered by the analysis included, among other things, information on the number of patients who made appointments in particular months, the number of first-time patients each month, the number of visits cancelled by patients each month, the number of patients who discontinued treatment each month, and patients’ preferences for a particular type of service in a given month. Comparisons were also made with regard to the impact of lockdowns on patients’ interest in dental services. The age and gender of the patients were also taken into account. The data were compared with those periods when lockdown restrictions were in place, and the possible impact of these lockdowns on patients’ interest in dental services was also analysed.

The lockdowns in Poland occurred during the following timeframes: March–April 2020, October–November 2020, December 2020–January 2021 and March–April 2021. Lockdowns included both nationwide and regional recommendations and restrictions regulating the movement of citizens, the presence of people in public buildings, the operation of individual services and the implementation of infection prevention and control guidelines. The above guidelines were recommendations.

2. The data were entered in an Excel file, cleaned, and then analysed. The results are summarized in summary tables and the data analysed.

Statistica 12 by StatSoft and StatXact by Cytel were used to calculated data. The significance level was determined α = 0.05. When *p* < α, the result was statistically significant. The Mann–Whitney test, the Student’s t-test and Shapiro–Wilk test were used. In order to test the relationship between categorical variables, the Chi2 test of independence, the Fisher exact test or the Fisher–Freeman–Halton test were calculated.

## 3. Results

In the conducted study, only for the parameter of cancelled visits and treatment continuation, the change in patient behaviour was statistically significant, while for the other measured parameters, the change in patient behaviour was not statistically significant.

During the lockdown period, there was a statistically significant decrease in the number of cancelled visits as compared to the year preceding the pandemic, *p* = 0.023. During the lockdown period, there was a statistically significant increase in the continuation of treatment by patients compared to the year preceding the pandemic, *p* = 0.01.

In the case of parameters such as first-time patients, the choice of the type of treatment and the type of dental services, including the reason and type of treatment, patients of particular age groups or gender of patients, there were no statistically significant changes in the pandemic period compared to the period preceding the pandemic.

Pearson’s r correlation was used to measure relation to the forecast of the number of habitants of Cracow and patients until 2040. Pearson’s r correlation is −0.417. The significance level is 0.485. The number of patients is not related to the projected population of the city of Cracow.

The results were analysed by comparing the percentage differences in the number of patients who made appointments in particular months during the pre-pandemic period with the number of appointments made during the COVID-19 pandemic, the percentage difference in the number of first-time patients in different months of the pre-pandemic period and during the pandemic, the percentage difference in the number of visits cancelled each month during the pre-pandemic period and during the pandemic itself, the percentage difference in the number of patients who discontinued treatment each month during the pre-pandemic period and during the pandemic itself, as well as the percentage difference with regard to patients’ preferences for particular services in a given month, in percentage terms, in the pre-pandemic period and during the pandemic itself. Comparisons were also made with regard to the impact of lockdowns on patients’ interest in dental services as well as in terms of the age and gender of patients.

**Figure 1:**
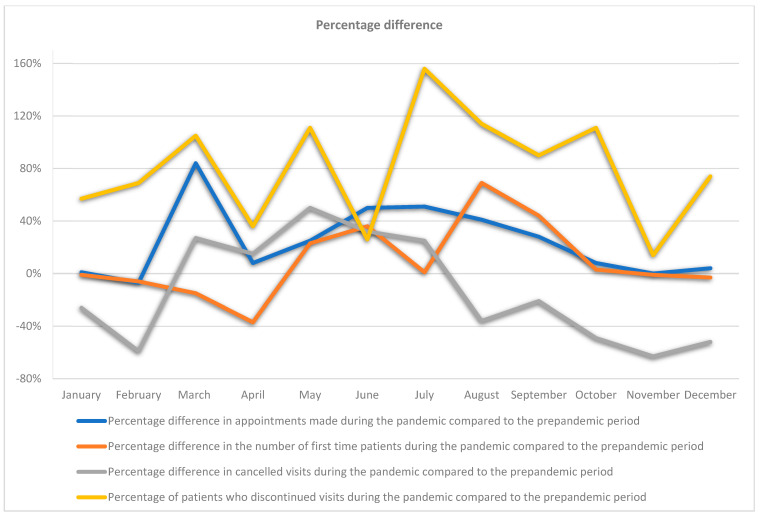
Percentage difference in the number of patients preferences each month both during the prepandemic period and during the pandemic itself.

During the COVID-19 pandemic from March 2020 to February 2021, compared to the year preceding the pandemic, that is, from March 2019 to February 2020, the number of visits arranged by patients in individual months, depending on the month studied, increased from 4% to 84%. There was only a decrease in the number of scheduled visits in February 2021 compared to February 2020 by 7%, and no changes in the number of scheduled visits in November 2020 compared to November 2019 (Figure 1). Differences in scheduled visits by patients in the period from March 2020 to February 2021 compared to the previous year, from March 2019 to February 2020, were not statistically significant.

The differences in the number of first-time visits in the pandemic year compared to the period before the pandemic, depending on the studied month, decreased from 1% to 37% for the studied months of March, April, November and December 2020, compared to March, April, November and December 2019 and for January and February 2021 compared to January and February 2020. The number of first-time appointments increased in May, June, July, August, September and October 2020 compared to May, June, July, August, September and October 2019. The increase in the number of first-time visits, depending on the surveyed month, ranged from 1% to 69% (Figure 1). Changes in the behaviour of patients in terms of first-time appointments were not statistically significant.

In the period of the COVID-19 pandemic from March 2020 to February 2021, compared to the period preceding the COVID-19 pandemic from March 2019 to February 2020, there was a statistically significant difference in the decrease in cancelled visits by patients *p* = 0.023. In the months of March, April, May, June and July 2020, compared to these months in 2019, there was an increase in cancelled visits from 15% to 50%. In the months from August 2020 to February 2021, there was a drop in cancelled visits from 21% to 63% compared to the period from August 2019 to February 2020 (Figure 1).

In the period from March 2020 to February 2021, compared to the period from March 2019 to February 2021, there was a statistically significant increase in the lack of continuation of treatment by patients, *p* = 0.01. The increase in the lack of continuation of treatment by patients, depending on the studied month, ranged from 26% to 156% in the period from March 2020 to February 2021, compared to March 2019 to February 2021 (Figure 1).

**Figure 2:**
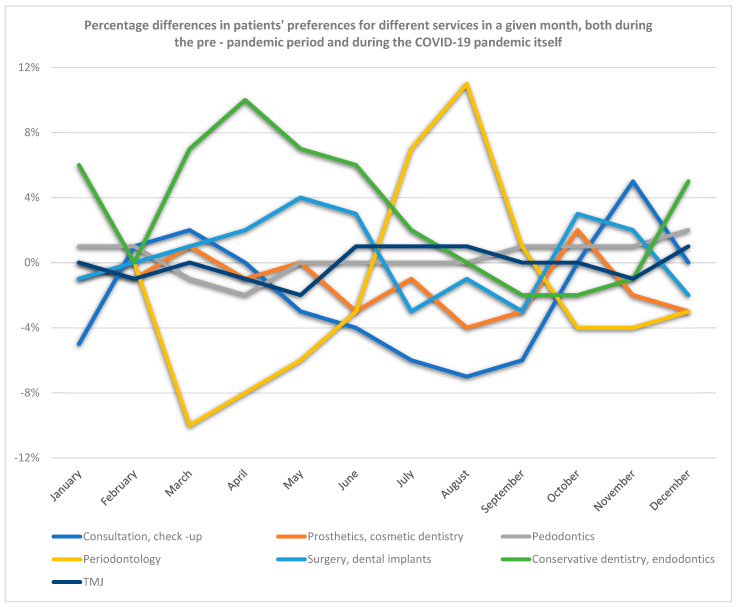
Percentage difference in patients’ preferences for different services in given month, both during the prepandemic period and during the pandemic itself.

During the COVID-19 pandemic from March 2020 to February 2021 compared to the previous year in the period from March 2019 to February 2020, there were no statistically significant differences in the treatment procedures performed by patients in individual dental specialties, i.e., in dental consultations, check-ups, prosthetic treatment, cosmetic dentistry, paediatric dentistry, periodontics, dental surgery, implantology, caries treatment, endodontics and treatment of the temporomandibular joint (Figure 2).

For dental services in the field of dental consultations, check-ups, dental prosthetics, pedodontics, dental surgery, implantology, and treatment of temporomandibular joint diseases, the number of services provided during the COVID-19 pandemic (March 2020 to February 2021) decreased up to 8% from the pre-pandemic period (March 2019 to February 2020). Only the number of services provided in the field of periodontics (treatment of gums and periodontitis) and treatment in the field of conservative dentistry (treatment of caries) and endodontics during the pandemic changed by 10% compared to the period before the pandemic (Figure 2).

**Figure 3:**
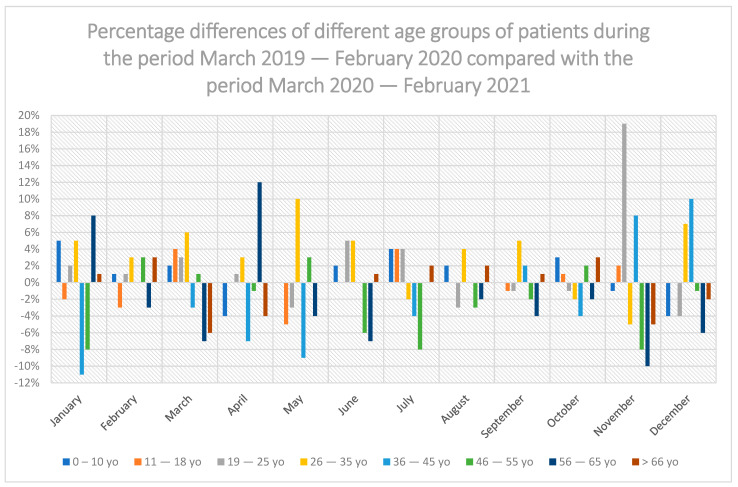
Percentage comparison of different age groups of patients during the period March 2019–February 2020 compared with the period March 2020–February 2021.

**Figure 4:**
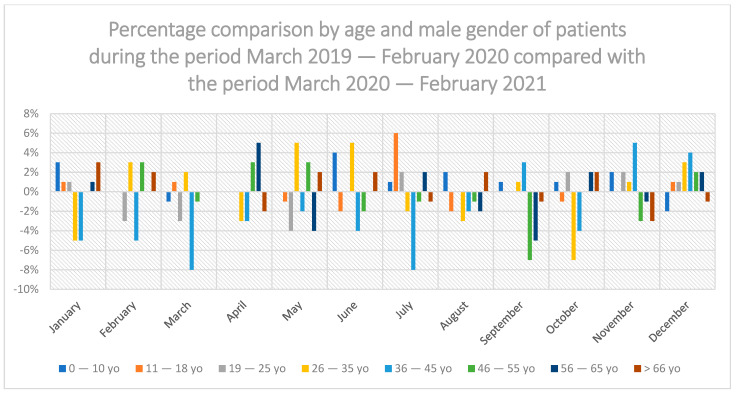
Percentage comparison by age and male gender of patients during the period March 2019–February 2020 compared with the period March 2020–February 2021.

**Figure 5:**
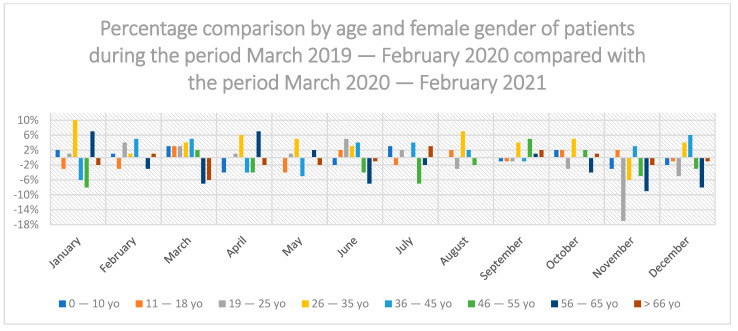
Percentage comparison by age and female gender of patients during the period March 2019–February 2020 compared with the period March 2020–February 2021.

In the period from March 2020 to February 2021 in relation to the period before the COVID-19 pandemic (March 2019 to February 2020), there was no statistically significant difference in the share of dental services in individual age groups: 0–10 years, 11–18 years of age, 19–25 years of age, 26–35 years of age, 36–45 years of age, 46–55 years of age, 56–65 years of age and >66 years of age (Figure 3). There was no statistically significant difference in both men (Figure 4) and women (Figure 5).

In the 19–25 age group, there was the highest increase in the use of dental services in relation to other groups in November 2020 by 19% compared to the previous year. The highest decrease in the use of dental services was recorded for the age group 36–45 in January 2021 by 11% compared to the previous year (Figure 3).

In the group of men during the COVID-19 pandemic, the highest decrease in the use of dental services was recorded in the group between 36–45 years of age in March and July 2020 by 8% compared to the previous year; the age group 11–18 among men recorded the highest increase in use of dental services in July 2020 by 6% compared to the year before the pandemic (Figure 4).

Among women, the highest increase in the use of dental services occurred in January 2021 in the age group 26–35 by 10% compared to the previous year; the highest decrease in the use of dental services occurred in November 2020 in the age group 19–25 by 17% compared to November 2019 (Figure 5).

**Figure 6:**
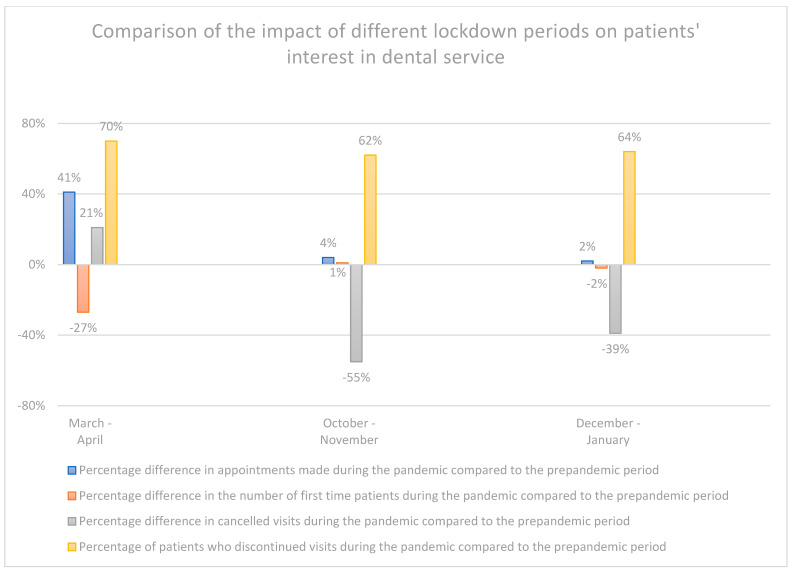
Comparison of the impact of different lockdown periods on patients’ interest in dental service.

During the lockdown periods, March–April 2020, October–November 2020, and December 2020–January 2021, compared to those periods in the previous year, there was an increase, depending on the lockdown period, from 62% to 70% of discontinuation of treatment by patients and an increase in the number of appointments by patients from 2% to 41%. In the period December 2020 to January 2021, compared to the previous year, there was a 2% decrease in first-time visits and a 39% decrease in the number of cancelled visits by patients. In the period March to April 2020, compared to the period March to April 2019, a 27% decrease was observed in first-time patients’ visits, with an increase by 21% of cancelled visits. In the period from October to November 2020, compared to this period in the previous year, there was an increase by 1% in the number of visits by first-time patients, with a decrease by 55% in the number of cancelled visits by patients (Figure 6).

## 4. Discussion

The COVID-19 pandemic changed patients’ perceptions of dental services and affected the functioning of existing dental offices [7,8,9,10,11,12,13], which is reflected both in patients’ preferences regarding dental services as well as in the type of patients seeking treatment, e.g., a decline in interest in dental services among new patients. This is confirmed by the results of the study.

By adapting to the new conditions of the pandemic and implementing the recommendations of the WHO and national organizations, dental offices were able to function safely [14,15,16,17,18,19,20].

To the author’s knowledge, few studies have been conducted that show patients’ preferences for dental services during the pandemic, much less compare these preferences with the pre-pandemic period [21,22,23,24]. The present study shows a number of indicators noted by dental offices regarding patient interest in their services. The study conducted is unique because it is the first to investigate the direct impact of the COVID-19 pandemic on patients’ preferences in choosing dental services. So far, studies have been carried out to determine the indirect impact on the behaviour of patients in dental services in the COVID-19 pandemic, and these studies analyse the impact of the COVID-19 pandemic on the behaviour of dental staff and, consequently, indirectly, on the preferences of patients [21,22,23,24]. The study eliminated the influence of factors such as the availability of personal protective equipment, which was freely available to the dental staff. The study was carried out directly in dental offices, and not through questionnaire inquiries to individual dentists, which provides direct access to detailed data, and thus a higher scope and detail of the study.

During the analysed periods, first-time patients’ interest in dental services decreased to 37% (in the month when interest in dental services was at its lowest) compared to the year preceding the COVID-19 pandemic. The number of cancelled visits rose by between 15% and 50% (depending on the analysed month) compared to the pre-pandemic period. During the pandemic, appointments by existing patients increased by up to 84% compared to 2019. During the pandemic, the percentage of patients who discontinued treatment rose by between 14% and 156% (depending on the analysed month) compared to the year preceding the pandemic. During the pandemic, patients’ preferences regarding dental services changed.

The recommendations of the WHO and the health organizations of individual countries regarding the functioning of health care have had an impact on patients’ preferences for particular dental office services, as a result of which interest in conservative dentistry and endodontics has increased, which in turn has resulted in more treatment of such conditions as carious cavities and pulpitis, i.e., pain relief, but also in a decline in interest in aesthetic dentistry. This is a natural phenomenon, which is probably a result of reduced social contacts, the introduction of social distancing rules, the obligation to cover one’s mouth in public, the introduction of remote working, and, hence, the absence of any need to show one’s smile, i.e., teeth in public. The lack of clear regulations regarding the functioning of dental offices may be responsible for the different ways dental offices operate in different countries of the world, which in turn has led to patients’ preferences varying from country to country [18,25,26,27,28,29,30].

According to the findings, the number of patients who made appointments in particular months of the pandemic increased compared to the pre-pandemic period, which may be a consequence of the recommendations governing the admission of patients to dental offices, i.e., a rotating system, longer breaks between patients and infection prevention and control guidelines, which translated into a limited number of visits to dental offices, and this in turn triggered greater interest among patients in making dental appointments.

An analysis of the differences in appointments made by first-time patients, i.e., patients who were making their first ever visit to a dental office, reveals a decrease in the number of appointments made during the lockdown period, i.e., March–April 2020 and December 2020–January 2021, which may be due to the fact that new potential patients were not aware of the infection prevention and control guidelines in force in a given dental office and were concerned about the risk of infection, which made them less inclined to book an appointment.

During the initial period of the pandemic, i.e., March to July 2020, there was a significant increase in cancelled visits compared to the year preceding the pandemic, while from August 2020 up until the end of the study, i.e., February 2021, the number of cancelled appointments declined. The likely reasons for the increase in cancelled visits during the initial months of the pandemic were patients’ fear of infection with SARS-CoV-2, and a lack of reliable information about the pandemic during the early months. The decline in the number of cancelled visits from August 2020 up until the end of the study in February 2021, a period characterised by lockdowns and a rising number of infections in the autumn of 2020, is difficult to explain. Most likely it was due to society’s fatigue with the restrictions introduced to combat the pandemic [31].

Throughout the entire period of the COVID-19 pandemic covered in the study, i.e., March 2020–February 2021, there was an increase in the number of patients discontinuing treatment. The likely reason for patients’ failure to continue dental care is the fact that during the COVID-19 pandemic, and in line with the recommendations introduced for dental settings, patients preferred emergency services, the purpose of which was to alleviate pain and protect the patient, and this in turn influenced the attitude of medical staff towards intervention services and patient preferences for oral health protection against pain. The most important factor shaping patients’ preferences for dental services was awareness campaigns aimed at encouraging the public to reduce social contacts and avoid leaving home for unnecessary reasons [31,32,33,34].

As regards age-related factors, no decrease was observed in the number of visits paid by patients aged 0 to 10 years during the COVID-19 pandemic compared to the preceding year. This correlates with the fact that there was also no decline in the number of dental visits made by patients aged 26–35 years, which is the age group comprising potential parents of children aged 0 to 10 years. The only period when the number of dental visits declined among patients aged 26 to 35 years during the year of the COVID-19 pandemic was during the second lockdown period from October 2020 until November 2020, which may have been due to an increase in COVID-19 infections in this age group during the autumn of 2020. The fact that patients from this age group continued to make dental appointments at the same rate as before the pandemic may be due to the fact that their main source of knowledge about COVID-19 is the Internet and social media, where it was possible to find contradictory information, often referred to as fake news, in which the existence and infectivity of the SARS-CoV-2 virus are sometimes questioned. Patients aged over 35 years, on the other hand, paid fewer dental visits compared with the year before the pandemic, especially during lockdown periods, i.e., March–April 2020 and the autumn of 2020, which may have been because of more information being obtained through television and the press and a greater respect for specialists and trust in medical experts. The number of dental visits made by patients aged 11–18 does not correlate with the group of potential parents, the reason perhaps being the independence of teenagers and their desire to make independent decisions [35,36,37,38,39,40,41].

Bearing in mind the potential further pandemics, it is worth adjusting the message to the recipient. In the case of younger age groups, i.e., under 35 years of age, the message, in addition to the specialist knowledge provided by scientists, should be influencers, i.e., social media stars on the Internet, in which this group trust [35,37,42,43,44].

In the case of patients over 35 years of age the message should be conveyed by specialists and scientists, and among other things highlight the safety of dental visits, bearing in mind the impact of oral health on the body as a whole and the risk of focal diseases, which in turn entails greater expenditure on treatment in the event of complications due to a lack of dental treatment or a delay in starting treatment [45,46].

As regards gender, a statistically significant decline was observed in the number of dental visits made by women aged 56–65 compared with men.

The main advantage of the study is the fact that it is one of the first research projects to analyse in detail patients’ preferences regarding dental services during the COVID-19 pandemic in comparison with the previous year. Epidemiological analysis of patients’ behaviour regarding their choice of dental services and oral health awareness plays a key role in preventing the focal diseases caused by poor oral health. Furthermore, the study analyses in detail the patients’ preferences for different dental specializations, the conduct of first-time patients and attitudes towards continuing treatment. Other studies, on the other hand, usually describe the impact of the COVID-19 pandemic on the functioning of dental offices and pay only marginal attention to patients’ preferences and choices of dental services during the COVID-19 pandemic [47].

This study is limited to an analysis of patients from five dental offices in the city of Cracow during the 12 months preceding the COVID-19 pandemic, i.e., from March 2019 to February 2020, as well as during the pandemic itself, from March 2020 until February 2021. Further long-term studies are recommended, based on patient data collected from the dental offices of various countries during the course of the entire pandemic as well as during the 12 months following the end of the pandemic.

What is important at the present time is to understand patient preferences for specific dental services, increase the number of patients making first-time appointments and reduce follow-up treatment during the COVID-19 pandemic. The pandemic lasted for more than one year and discontinuing dental services for such a long period of time is dangerous for patients’ health, as such behaviour is associated with the emergence and exacerbation of focal diseases that affect the entire body. Neglecting treatment can also result in more acute oral problems, which in turn require more expensive, long-term treatment. This may not only lead to the deterioration of the patient’s health, but also to the need for higher financial outlays. In view of the prospect of future pandemics, it is vital that procedures are implemented that prevent neglect of oral health and the subsequent risk of focal diseases [48,49].

## 5. Conclusions

Further long-term research is needed to help gather relevant data that can be used as the basis for protocols and recommendations for dental office visits during a potential pandemic. These should ensure that visits are safe for both patients and staff and have the health of patients in mind. Protocols and recommendations for patients in each age group should be channelled through different media sources.

## Data Availability

Non-digital data supporting this study are curated by Klaudia Migas.
